# Process modeling and sludge characterization of electrocoagulation for the removal of oil-in-water emulsions and calcium from petroleum refinery wastewater

**DOI:** 10.1038/s41598-026-37854-8

**Published:** 2026-03-03

**Authors:** Yomna E. Mohamed, Dina A. El-Gayar, Nevine K. Amin, E.-S. Z. El-Ashtoukhy

**Affiliations:** Chemical Engineering Department, Faculty of Engineering, Alexandria University, Alexandria, Egypt

**Keywords:** Response surface methodology, Optimization, Operating cost, COMSOL simulation, Chemistry, Energy science and technology, Engineering, Environmental sciences

## Abstract

Electrocoagulation (EC) process efficiency for treating synthetic petroleum refinery wastewater was investigated. The novelty of this study lies in the simultaneous removal of oil-in-water emulsion and calcium ions using an integrated experimental–statistical–financial approach, combining response surface methodology (RSM) optimization with COMSOL Multiphysics simulation. The impact of independent variables on the removal rates of both contaminants was studied and optimized using the central composite design method. Analysis of variance was employed to evaluate the significance of the variables and the mathematical model determined by RSM. The optimal conditions were determined to be a pH of 9, a current density of 6.123 mA/cm^2^, an initial calcium concentration of 130 ppm, an initial oil content concentration of 588 ppm, a NaCl concentration of 2.5 g/l, and a total electrolysis time of 98 min. These conditions correspond to an oil content removal rate of 91.3% and a calcium removal rate of 72.9%. Energy consumption and total operation costs were calculated under these parameters to be 12 kWhm^-3^ and 10.32 EGPm^-3^, respectively. Fourier Transform Infrared spectroscopy, Energy-dispersive X-ray spectroscopy, and Scanning electron microscopy characterization were performed on the resulting sludge and scum at the optimum conditions and its utilization was discussed. Furthermore, COMSOL Multiphysics software was used to simulate the voltage distribution across the proposed cell to understand the electrochemical features. Overall, the statistical, financial, and simulated study demonstrates the feasibility of the EC technique for oil refinery wastewater treatment.

**Supplementary Information:**

The online version contains supplementary material available at 10.1038/s41598-026-37854-8.

## Introduction

From crude oil, the petroleum refining sector creates more than 2,500 refined goods, including liquefied petroleum gas, gasoline, kerosene, diesel fuel, and lubricating oils^1^. For cooling, cracking, desalting, distillation, hydrotreating, and stripping steam, petroleum refineries use enormous amounts of water^2^, hence producing excessive volumes of petroleum refinery wastewater (PRW). In 2023, Egypt’s total liquid fuels production reached 610,000 barrels per day (b/d), with around 565,000 b/d comprising crude oil and lease condensate^3^. According to Ezugbe et al.^4^, the processing of each barrel of crude oil results in the generation of about ten barrels of petroleum wastewater. Moreover, literature research has confirmed that about 1.6 times the volume of refined crude oil is produced as wastewater effluent^5–7^.To process one barrel of crude oil, a minimum of approximately 60–90 gallons of water is required^4^. Therefore, this enormous volume of PRW effluents is always generated and dumped into the primary water bodies on Earth. Consequently, before release into the open environment, PRW must be managed to meet a particular quality criterion. PRW may include biological oxygen demand (BOD) and chemical oxygen demand (COD) levels between 150 and 350 mg/l and between 300 and 800 mg/l, respectively, phenols content between 20 and 200 mg/l, oil content up to 3000 mg/l, suspended solids over 100 mg/l^8^ and total hardness ranges between 120 and 480 ppm^9,10^. Oil content contained in PRW typically exists in four forms: free, dispersed, emulsified, and dissolved oil^11^ and may contain diesel oil, grease, or lubricating oil^12,13^. This oil contaminates the sewage system, lowers the concentration of dissolved oxygen by causing bacterial oxidation of organic material, inhibits the formation of algae, and alters the flavor, odor, and color of water resources^14^. PRW frequently contains calcium and magnesium salts. The presence of these substances might result in scaling issues in the API oil separators and other equipment that are often used in the refinery^15^. The cost of water disposal can be lowered, and potential negative environmental effects can be lessened by properly treating PRW for advantageous recycling and reuse. In refineries, the conventional effluent treatment process is based on mechanical and physicochemical methods such as mechanical oil–water separation (sedimentation and flotation) and chemical process (coagulation–flocculation), which are followed by biological treatment. Numerous studies have been conducted to develop effective methods for treating PRW. Among the methods employed are membrane technologies^16^, biological treatment^17^, chemical oxidation^18^,and coagulation and flotation^19^. Electrochemical technologies—mainly electrocoagulation (EC), electrooxidation (EO), electro-Fenton (EF), and electrodialysis (ED)—are increasingly being applied to treat petroleum industry effluents as robust alternatives to conventional methods^20^. EO uses conductive anodes to generate hydroxyl radicals and other oxidizing species, which break down tough, persistent organic pollutants like dyes and pharmaceuticals. EF generates electrochemically produced gas microbubbles to float emulsified oils and colloids, often outperforming conventional dissolved air flotation^21^. ED provides ion-selective separation, ideal for desalination and treating saline or ion-rich wastewaters^20^. In contrast, EC stands out as the most extensively utilized electrochemical method, applied at both bench- and pilot-scale levels, for treating various effluents from the petroleum sector^22^. This is because EC has been suggested as a substitute for conventional chemical coagulation. EC has been demonstrated to be effective at eliminating colors and dyes^23^, suspended solids^24^, oil and greases^12^, heavy metals^25^, fluoride^26^, as well as hardness^26^. Furthermore, EC has demonstrated outstanding efficiency as a pretreatment step before reverse osmosis, successfully removing various pollutants and lessening membrane fouling^15,27^. However, in comparison to membrane technology, which is an energy-intensive technology^28^, the inlet water composition in EC does not necessitate thorough pretreatment to eliminate particulates^29^. In addition, under an electric flux, the EC is capable of destroying bacteria by rupturing cells^30^. Since there is minimal enrichment of ions in solutions, the scum formed during EC consists of lightweight flocculated particles. The produced hydrogen and oxygen bubbles at the cathode and anode, respectively, are essential for promoting its formation by buoying flocculated contaminants to the water surface, which improves their removal^31^. Furthermore, the daily procedure for EC technology is simple, and it is not complicated when compared to membrane or electrodialysis techniques. Thus, EC methods are simpler, more reliable, chemical-free, and more affordable than other technologies^29^. EC does not require external coagulants because they are produced in situ by dissolving metal anodes. Aluminum and iron are frequently used electrodes. The Al electrodes used in EC cells are more advantageous than Fe electrodes for many reasons. Fe corrodes in water spontaneously, regardless of the current flux, resulting in uncontrollable production of Fe-derived coagulants. The Al electrodes, on the other hand, go through surface passivation by creating an Al_2_O_3_ layer, and unlike Fe^3+^, the Al^3+^ can regulate under a regulated current flux, the Al^3+^ polymerizes to form (Al(OH)^2+^, Al(OH)_2_^+^, Al_2_(OH)_2_^4+^) and poly-nuclear species (Al_6_(OH)_15_^3+^, Al_7_(OH)_17_^4+^, Al_8_(OH)_20_^4+^, Al_13_(OH)_34_^5+^, Al_13_O_4_(OH)_24_^7+^), which are eventually turned into aluminum hydroxide: Al(OH)_3_. The large specific area of Al(OH)_3_ then facilitates compound adsorption and traps the colloids^29^. Therefore, in this study, an Al sacrificing anode is used to examine dual oil content and CaCl_2_ removal from water. Equation (1–3) shows the actual reactions taking place inside the cell^32^.

1 $$Al \to Al^{3 + } + 3e^{ - }$$

2 $$3H_{2} O + 3e^{ - } \to H_{2} + 3OH^{ - }$$

3 $$Al^{3 + } + 3H_{2} O \to Al\left( {OH} \right)_{3} + 3H^{ + }$$

The ability of EC to eliminate hardness/calcium and magnesium salts^26,33,34^ and oil-in- water emulsions^12,35,36^ has been examined separately in several studies. However, the simultaneous removal of these contaminants has received limited attention in the existing literature. Sefatjoo et al.^27^ studied the impact of initial calcium concentration, initial turbidity, operation time, and current density on the turbidity, calcium removal rates, and the operating cost, where the EC process was used as a pretreatment step to the reverse osmosis technique. In addition, Gonçalves et al.^37^ found the influence of pH, working temperature, and EC time on the removal efficiency of calcium, sodium, strontium, and COD reduction. Finally, the consequence of changing pH, electrolysis time, and current density on the removal of hardness, turbidity, and COD reduction from a real produced water were discussed by Zhao et al.^15^. However, most existing EC studies focus on the removal of a single pollutant, either calcium ions or oil-in-water emulsions, and do not address their simultaneous removal from refinery wastewater. In addition, previously mentioned research has typically studied a limited number of operating parameters and did not combine statistical design tools with electrochemical modeling.

The present study aims to bridge this research gap by investigating the influence of the six independent variables (electrolysis time, pH, current density, concentration of electrolyte, which is sodium chloride (NaCl), initial calcium concentration, and initial oil content concentration) on the removal rates of both calcium and oil content and find out the interactions between them. The study effectively utilizes Design-Expert software to apply RSM for optimizing the independent variables. The central composite design (CCD) approach is employed to establish the required number of experimental runs for optimizing both the process parameters and the corresponding responses, as well as to identify the optimal conditions, and to find out the mathematical correlation between them. Statistical analysis with regression and analysis of variance (ANOVA) is carried out on experimental results**.** In addition, the commercial feasibility of the technique is determined by calculating energy consumption and the total operating cost. Moreover, COMSOL Multiphysics software is used to generate visual simulation and investigate the potential distribution within the proposed cell, thus revealing its electrochemical properties.

## Materials and methods

### Preparation of synthetic solution

In this study, synthetic wastewater with varying initial concentrations of oil content and calcium was prepared to simulate real wastewater conditions. Solution 1 was prepared by dissolving the required concentration of lubricating oil (SAE 20 W / 50 MISR 1 Engine Oil) in 1 mL of the emulsifier Tween 80. The characteristics of the lubricating oil are illustrated in Table 1. Solution 2 was prepared by dissolving the specified amounts of CaCl₂ and NaCl, which was added to enhance the conductivity of the solution, in 200 mL of distilled water. The pH of solution 2 was then adjusted using either 10% sodium hydroxide (NaOH) or 10% hydrochloric acid (HCl) solutions.

**Table 1:** General characteristics of the lubricating engine oil.

Parameter	Value
SAE viscosity grade	20 W / 50
Performance Level	API SL / CF
Density @ 35 °C	0.885 g/ml
Kinematic viscosity @ 40 °C	166 mm2/s
Kinematic viscosity @ 100 °C	19 mm2/s
Viscosity Index	127
Pour Point	-27 °C

Subsequently, solutions 1 and 2 were combined and stirred at room temperature at 1000 rpm for 1 h. Finally, distilled water was added to bring the total volume to 2 L, and the final mixture was stirred for an additional 15 min. All chemicals employed in this study were of analytical grade.

### Electrocoagulation system

**Fig. 1:**
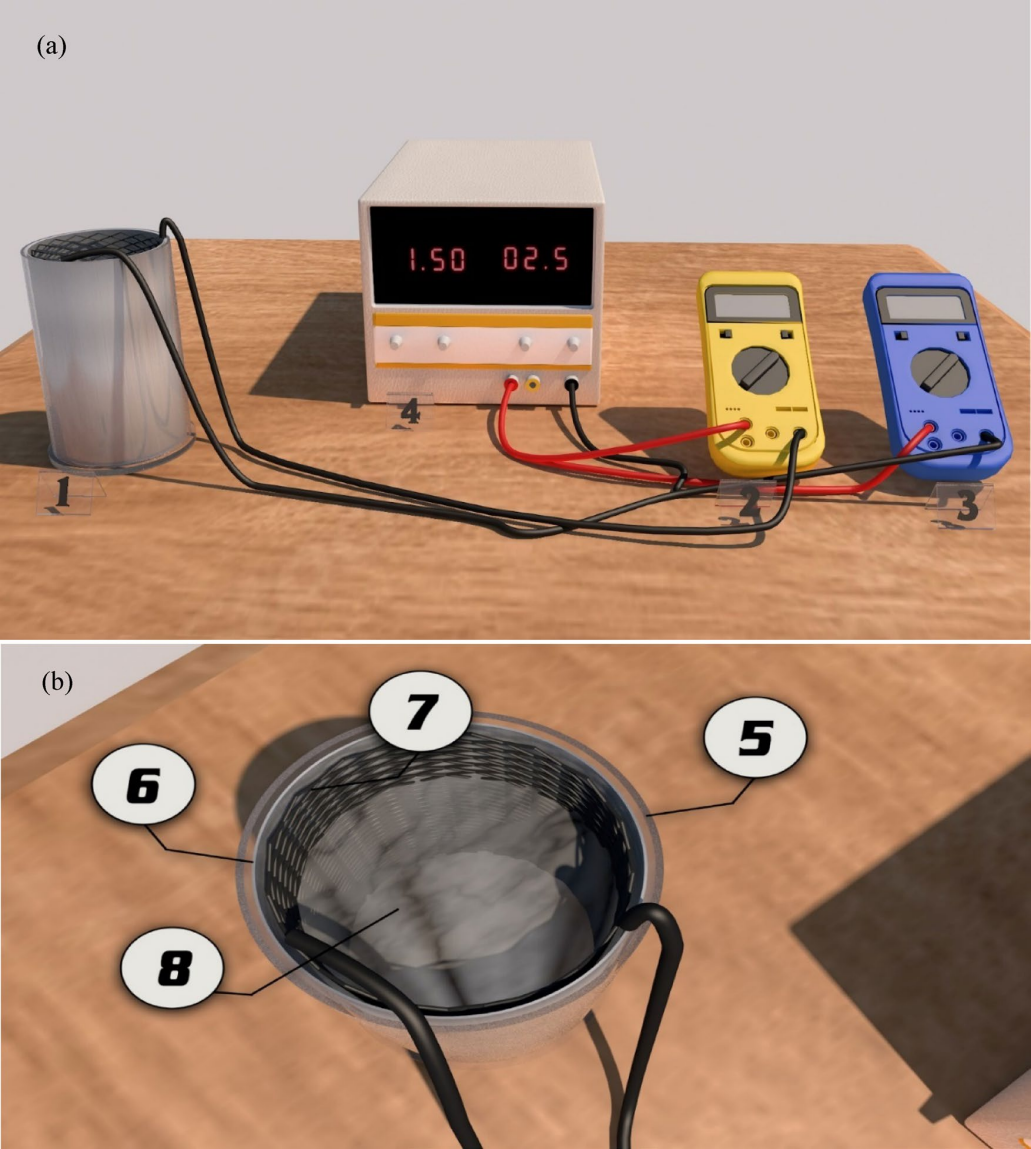
(**a**) Photographic view of the EC experimental setup, including (1) EC reactor, (2) Ammeter, (3) Voltmeter, and (4) DC Power supply. (**b**) Schematic diagram of the EC cell illustrating the main components: (5) Plexiglass cylinder (6) Anode (7) Cathode (8) Synthetic Solution.

Figure 1a illustrates the setup of the EC cell used in the present study. Experiments were performed in a cylindrical-shaped plexiglass reactor, which is 15 cm in diameter and 25 cm in length. The anode is an aluminum cylindrical sheet lining the plexiglass reactor as shown in Fig. 1b. The cathode is a cylindrical aluminum screen located 5 mm from the anode.

The ZHAOXIN RXN-305D DC power supply was used to supply direct current. The cell and a digital ammeter were connected in series with the power source and a digital voltmeter was connected in parallel with the cell. Experiments were carried out using a constant working volume of 2 L and at room temperature. The electrochemical reactor was stirred by H_2_ bubbles produced at the cathode to minimize mass transfer constraints^38^. The electrodes were submerged in 5% HCL for 5 min before each experiment to eliminate any passivating substance. Then, they were rinsed with distilled water and left to dry.

### Analytical methods and calculations

Calcium and oil content removal efficiencies were calculated to evaluate the performance of EC technique. Oil content concentration was measured using a UV–visible Spectrophotometer. According to Beer–Lambert law, the percentage of oil content removal was measured according to the following Eq. (4)^39^.

4 $$\%{R}_{1}=\frac{{A}_{0}- {A}_{f}}{{A}_{0}}*100$$

where ${A}_{0}$, ${A}_{f}$ represents the initial and the final oil content absorbance, respectively. ${R}_{1}$ represents the removal percentage of oil content.

Calcium ion concentration was measured using the EDTA titration method^40^. The percentage removal of calcium is calculated according to Eq. (5).

5 $$\%{R}_{2}=\frac{{C}_{0}- {C}_{f}}{{C}_{0}}*100$$

where ${C}_{0}$ is the initial calcium concentration, ${C}_{f}$ is the final calcium concentration, and ${R}_{2}$ is the removal percentage of calcium.

The electrical energy consumption (E) (kWhm^-3^) of the treated water was calculated using Eq. (6)^41^.

6 $$E=\frac{VIt}{60*Vol}$$

where t denotes the operating time in minutes, V signifies the voltage of the cell in volts, I is the applied current in amperes, and Vol represents the volume treated in liters.

The total operating cost (TOC) of the EC process includes the cost of the energy consumed (E), the consumption of the electrode usage (CEU), chemical consumption (to enhance electrical conductivity or adjust pH), and the cost of sludge treatment^42^. Due to their most obvious impact, the current study only considered the expense of energy consumption and the cost of the working electrodes. The TOC can be calculated from Eq. (7–9)^43^.

7 $$TOC\left( {EGP/m^{3} } \right) = a*E + b*CEU$$

8 $$CEU (gm Al/m3) =\frac{\omega *1000}{Vol}$$

TOC is the Total Operating Cost (*EGP/m*^*3*^), CEU represents the electrodes consumption (*gm Al/m*^*3*^), a is the cost of 1kWh energy consumption (*EGP* /kWh), and b is the cost of 1 gm Al plate (*EGP* /gm).

$\omega$ (gm Al) is the theoretical maximum possible mass of Al produced from the anode, obtained from Faraday’s law (Eq. 9)^44^.

9 $$\omega =\frac{IMt*60}{nF}$$

where M stands for the molecular weight of Al = 27 $\frac{gram}{mole}$, n is the number of free electrons of Al in every transfer = 3, and F represents Faraday’s constant = 96,485 $\frac{Col}{mole}$.

In July 2025, the values of a = 0.85 piasters (*EGP* /kW.h) and b = 0.123 (EGP/gm) for Al, respectively.

The geometric surface area of the cylindrical anode in contact with the solution was used for current density calculations and was determined according to:

10 $$A=\pi dL$$

where *d* is the diameter of the cylindrical anode (14 cm) and *L* is the effective height of the electrode immersed in the solution (13 cm). Only the immersed surface area was considered. The applied current density (J) was calculated as:

11 $$J=\frac{I}{A}$$

where *I* is applied current.

### Experimental design and model development

Design-Expert software version 13.0.5.0 (Stat-Ease, Inc., Minneapolis, USA) was used to optimize the independent variables. Specifically, CCD was employed to identify the significant parameters, evaluate the relationships between the variables and the responses, and determine the optimal operating conditions. The ranges of independent variables are listed in Table 2.

**Table 2:** Range of the independent parameters.

	Parameter	Unit	Minimum	Maximum	Coded Low	Coded High	Center point
A	pH	–	5	10	-1 ↔ 6.00	+ 1 ↔ 9.00	7.50
B	Current density	mA/cm^2^	1.75	7	-1 ↔ 2.63	+ 1 ↔ 6.13	4.37
C	Initial oil content concentration	ppm	50	800	-1 ↔ 185.00	+ 1 ↔ 665.00	425
D	Initial calcium concentration	ppm	50	500	-1 ↔ 130.00	+ 1 ↔ 420.00	275
E	NaCl concentration	g/l	2	6	-1 ↔ 2.50	+ 1 ↔ 5.50	4
F	Electrolysis time	min	0	120	-1 ↔ 22.00	+ 1 ↔ 98.00	60

The variables were studied at five levels (+ α, + 1, 0, -1, α). For the axial points, the + α coded levels correspond to the following actual values:

**Table:** 

pH: + α = 10	Current density: + α = 7 mA/cm^2^
Initial oil content concentration: + α = 800 ppm	NaCl concentration: + α = 6 ppm
Initial calcium concentration: + α = 500 ppm	Electrolysis time: + α = 120 min

The range of these parameters was identified based on literature review^10,37,45^. Design Expert provides efficient design of 84 experiments shown in supplementary data (Supplementary Table S1).

The relation between the variables and the responses, in many cases, is described using a second-order polynomial equation. The general expression can be represented in Eq. (12)^46^.

12 $$Y = \beta_{0} + \mathop \sum \limits_{i = 1}^{n} \beta_{i} x_{i} + \mathop \sum \limits_{i = 1}^{n} \beta_{ii} x_{ii}^{2} + \mathop \sum \limits_{i < j}^{n} \sum \beta_{ij} x_{i} x_{j} + \varepsilon$$

where Y is the response, ${\beta }_{0}$ is constant, ${\beta }_{i}$ is the linear effect coefficient for the i^th^ factor, ${\beta }_{ii}$ is the quadratic effect coefficient for the i^th^ factor, and ${\beta }_{ij}$ is the interaction effect coefficient for i^th^ and j^th^ factors and ε is the random error. The least-squares method is employed to fit the model with experimental data.

### Simulation EC process with COMSOL software

The electrochemical module of COMSOL Multiphysics software version 6.2 (COMSOL, Inc., Burlington, MA, USA) was used to simulate and illustrate the potential distribution within the cell. In theory, the fundamental need for addressing the issue is to develop the geometry of the used EC cell, then identify the governing equations.

#### Governing equations

The current distribution equation is used for applied voltage.

13 $$For \, electrolyte:\nabla \cdot i_{l} = Q_{l} ,\;i_{l} = - \sigma_{l} \nabla \varphi_{l}$$

14 $$For\;electrodes:\nabla \cdot i_{s} = Q_{s} ,\;i_{s} = - \sigma_{s} \nabla \varphi_{s}$$

whereas σ_l_ and σ_s_ are the electrolyte and electric conductivity (S/m) respectively, φ_l_ and φ_s_ are the electrolyte and electric potential (V). Q represents the additional charge.

##### Situation of the boundary

The boundary of the external electric potential at the anode and cathode is:

At anodes: φ_s,ext_ = E_cell_.

At cathodes: φ_s,ext_ = 0.

## Results and discussions

### Oil content removal rate experimental analysis

#### Fitting the model

A response surface regression method was used to fit a polynomial model to the experimental data. The statistical program carried out ANOVA for the model. Among linear, two-factorial, quadratic, and cubic models, the quadratic model was statistically significant (*p*-value < 0.0001). The ANOVA results for the quadratic model are represented in Table 3. The higher value of R^2^ (0.9347) and adjusted R^2^ (0.9033) show that the observed response and predicted model have adequate compatibility. The predicted R^2^ of 0.8436 is in reasonable agreement with the adjusted R2 of 0.9033, as the difference is less than 0.2.

**Table 3:** ANOVA for quadratic model for % Removal of oil content.

Source	Sum of squares	df	Mean square	*F*-value	*p*-value	
Model	18,570.10	27	687.78	29.70	< 0.0001	Significant
A-pH	17.04	1	17.04	0.7358	0.3947	
B-Current density	799.91	1	799.91	34.54	< 0.0001	
C-Initial oil content conc	708.91	1	708.91	30.61	< 0.0001	
D-Initial calcium conc	733.19	1	733.19	31.66	< 0.0001	
E-NaCl	63.51	1	63.51	2.74	0.1033	
F-Electrolysis time	7528.34	1	7528.34	325.12	< 0.0001	
AB	1.85	1	1.85	0.0799	0.7785	
AC	6.47	1	6.47	0.2795	0.5991	
AD	120.10	1	120.10	5.19	0.0266	
AE	26.72	1	26.72	1.15	0.2874	
AF	16.59	1	16.59	0.7166	0.4009	
BC	437.68	1	437.68	18.90	< 0.0001	
BD	7.26	1	7.26	0.3133	0.5779	
BE	8.67	1	8.67	0.3742	0.5432	
BF	1738.35	1	1738.35	75.07	< 0.0001	
CD	67.57	1	67.57	2.92	0.0931	
CE	0.9203	1	0.9203	0.0397	0.8427	
CF	46.99	1	46.99	2.03	0.1598	
DE	94.75	1	94.75	4.09	0.0479	
DF	8.87	1	8.87	0.3829	0.5386	
EF	41.98	1	41.98	1.81	0.1836	
A^2^	586.76	1	586.76	25.34	< 0.0001	
B^2^	657.33	1	657.33	28.39	< 0.0001	
C^2^	10.83	1	10.83	0.4677	0.4969	
D^2^	544.47	1	544.47	23.51	< 0.0001	
E^2^	2167.14	1	2167.14	93.59	< 0.0001	
F^2^	2044.82	1	2044.82	88.31	< 0.0001	
Residual	1296.72	56	23.16			
Lack of Fit	1172.90	47	24.96	1.81	0.1708	not significant
Pure Error	123.82	9	13.76			
Cor Total	19,866.82	83				

The Adeq Precision in this model is equal to 31.741, which measures the signal-to-noise ratio. A ratio greater than 4 is desirable. This indicates that the model gives reasonable performance in prediction.

**Fig. 2:**
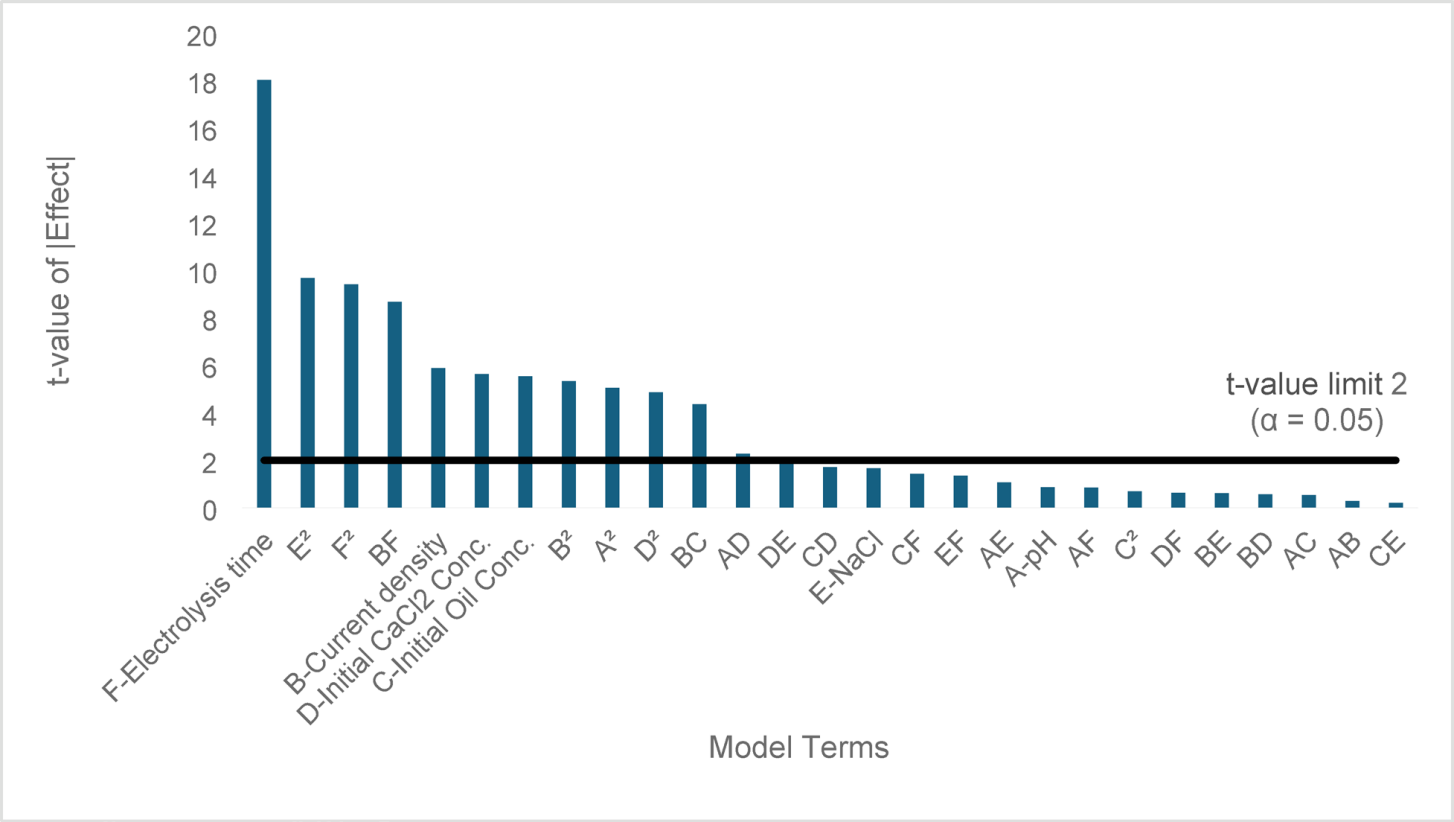
Pareto chart of standardized effects for oil content removal rate. The horizontal reference line represents the statistical significance threshold at α = 0.05 (t = 2). Model terms with standardized effects exceeding this line are statistically significant.

The Lack of Fit *F*-value of 1.81 implies that the Lack of Fit is not significant relative to the pure error. The important thing is that the non-significant lack of fit is good for the proposed model. The *p*-values were used to determine the statistical significance of the equation’s terms and to reflect the interaction intensity between each independent parameter. *P*-values below 0.05 indicate model terms are significant. Here, the key variables in this model are B-current density, C- initial oil content concentration, D- initial calcium concentration, F- electrolysis time, AD, BC, BF, DE, A2, B2, D2, E2, and F2 are significant model terms. Values greater than 0.1 can be removed from the model because these terms are insignificant. Figure 2 shows the Pareto chart for the independent variables, showing that the electrolysis time is the most crucial variable. Thus, the relation between the oil content removal percentage in terms of the actual values of the investigated parameters is given by Eq. (15)

15 $$\begin{gathered} \% \;Removal\;of\;oil\;content = 297.22426 \, - \, 36.41198A \, - \, 26.35613B \, - 0.008880C \, + \, 0.159489D \hfill \\ - 56.73242E \, + \, 0.968748F \, + \, 0.066209AB \, + \, 0.000903AC \, - \, 0.006439AD \hfill \\ - \, 0.294155AE - 0.009151 \, AF \, + \, 0.006366BC \, - \, 0.001357BD \, - \, 0.143604BE \hfill \\ + \, 0.080287BF \, + \, 0.000030CD \, - \, 0.000341CE - 0.000096CF \, + \, 0.005731DE \hfill \\ + \, 0.000069DF \, + \, 0.014526 \, EF \, + \, 2.63815 \, A^{2} + \, 2.43240 \, B^{2} - \, 0.000016 \, C^{2} \hfill \\ - \, 0.000306 \, D^{2} + \, 7.07554 \, E^{2} - \, 0.008391 \, F^{2} \hfill \\ \end{gathered}$$

#### Influence of the operating parameters

3D surface plots are used to discuss the influence of the operating parameters as well as their interactions to help understand and optimize the model. Each of the 2 factors is plotted while keeping the other factors at the center points.

##### Electrolysis time/current density

**Fig. 3:**
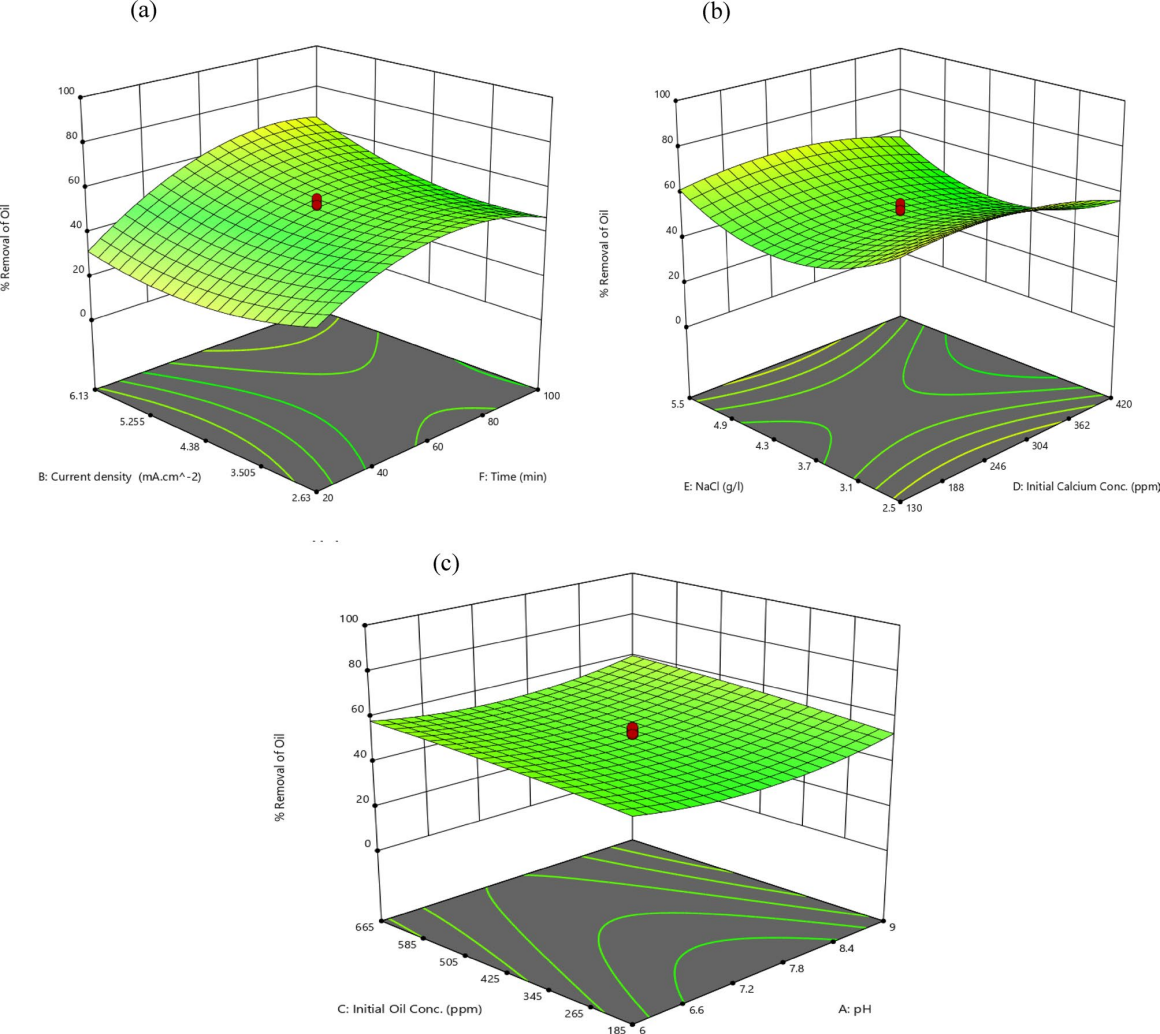
3D surface plots for oil content removal rate (**a**) electrolysis time and current density (**b**) Initial concentrations of both calcium and NaCl (**c**) Initial concentration of oil and pH.

Figure 3a shows the influence of both current density and electrolysis time on oil content removal rate. Extending the duration from 22 to 98 min results in a 28.3% increase in the oil content removal rate, from 37.65% to 48.3%, at a constant current density of 2.63 mA/cm^2^. When the current density reaches 6.13 mA/cm^2^, increasing the electrolysis time further enhances the removal rate more consistently, by 94%, from 34 to 66%.

According to Faraday’s law (Eq. 9), the beneficial effect of raising the oil content removal rate with electrolysis time is that it produces more of the adsorbent aluminum hydroxide flocs. Additionally, more gas bubbles are created, which facilitate the transport of destabilized pollutants to the solution’s surface through flotation and aid in their removal^47^.

The impact of current density on the removal rate follows an unsteady pattern, with minor variations observed in this research.

At a short electrolysis time interval (22 min), increasing the current density from 2.63 mA/cm^2^ to 4.37 mA/cm^2^ reduces the removal rate from 37.65% to 28.36%, while raising the current density further to 6.13 mA/cm^2^ boosts the removal rate once more, up to 34%.

Insufficient electrolysis time may cause this, since the produced metal hydroxides are insufficient to adequately destabilize the oil droplets, and at this unstable level, the rapid increase in current density results in additional convection currents^48^ which disturp the productions of the flocs, and the consistent distribution of the reactants at the electrode^49,50^.

However, a further increase in the current density enhances the formation of the hydroxides, causing better removal rates, but not as high as the initial values. Beyond an optimum current density, further increase lead to higher energy consumption and operating cost.

Similar findings were reported by Merzouk et al.^48^ that the turbidity removal rates were negative at high current densities and short treatment time. Sefatjoo et al.^27^ also discovered an adverse effect of increasing the current density ( 3.85 mA/cm^2^ to 7.15 mA/cm^2^) on the turbidity removal rate, which was carried out at low periods (22.5 min and 47.5 min).

Controversially, at higher electrolysis time periods = 98 min, raising the current density from 2.63 mA/cm^2^ to 6.13 mA/cm^2^, increases the removal rate from 48.3% to 66%. This trend is familiar in literature^51,52^ following Farady’s law. Moreover, the increased production of hydrogen gas bubbles causes simultaneous reduction in their size, which improves solution mixing and speeds up the removal rate^53^. The uniform distribution of reactants at higher current densities and voltage is simulated in section “COMSOL Simulation”. to illustrate these findings.

##### Initial concentrations of both calcium and NaCl

As depicted in Fig. 3b, the initial concentration of calcium affects the removal rate similarly when the NaCl concentration is held constant at either 2.5 g/l or 5 g/l. Initially, increasing the calcium concentration from 130 to 275 ppm results in a minor enhancement in the removal rate, approximately 5%. This is attributed to the rise in the solution’s conductivity, which consequently reduces its electrical resistance and improves the removal efficiency^54^.

However, a further increase in concentration up to 420 ppm lowers the removal rate by 16% and 13% at NaCl concentrations of 2.5 g/l or 5 g/l, respectively. The main reason is that calcium is removed by forming an insulating layer of calcium carbonate at the cathode as in Eq. (16–17) when the pH is high^55^. The formed insulating layer steeply raises the potential difference between the electrodes, causing a significant drop in the current efficiency^56^.

16 $$HCO_{3} + \, OH^{ - } \to \, H_{2} O \, + \, CO_{3}^{2 - }$$

17 $$CO_{3}^{2 - } + \, Ca^{2 + } \to CaCO_{3}$$

Concerning the effect of NaCl concentration, according to Fig. 3b, the removal rate drops by roughly 27% when the concentration of NaCl is increased from 2.5 g/l to 4 g/l, then raising it to 5.5 g/l steps up the removal rate to reach 61.73% at an initial calcium concentration of 130 ppm.

The initial negative impact is attributed to the fact that electrophoresis reduces the demulsification by increasing NaCl concentration since the competing chloride preferentially migrates to the anode surface over the initially negatively charged oil droplets^57^. However, a further increase in the electrolyte concentration improves the conductivity and the generated current in the electrolysis cell, which in turn causes more metal hydroxides to form and more oil droplets to adsorb^58^. Additionally, the passivation layer that forms on the electrode surface as electrolysis proceeds is also removed by the rise in Cl^-^ ions formed by NaCl. Chlorine currently functions as the antipassive agent that breaks down the Al_2_O_3_ oxide film coating the Al anode and prevents it from dissolving into Al^3+^^59^. As a result, there are more coagulants available in the solution.

The electrolysis of chloride salts results in the formation of molecular chlorine:

18 $$2Cl^{ - } \to Cl_{2(g)} + 2e^{ - }$$

The generated molecular chlorine is hydrolyzed to hypochlorous acid and hypochlorite ions as in Eq. (19–20). Owing to their high oxidative potential, these species are responsible for removing pollutants^60^. Furthermore, chloride ions greatly lessen the adverse impact of the insulating layer on the electrodes’ surface caused by the precipitation of CaCO_3_^56^.

19 $$Cl_{2(g)} + H_{2} O \, \to \, HOCl + \, H^{ + } + \, Cl^{ - }$$

20 $$HOCl \to ClO^{ - } + H^{ + }$$

To conclude, the presence of both calcium and NaCl influences the removal rate, but they must be kept at an optimum value to achieve the best efficiency.

##### Initial oil content concentration/pH

Figure 3c shows the positive impact of elevated initial oil content concentration on the removal rate.

The removal rate is enhanced by 11% and 13.6% when oil content concentration steps up from 185 to 665 ppm at constant pH of 6 and 9, respectively. This finding is supported by previous research^56,61^. The primary cause is that as the initial oil content concentration rises, the collision frequency of the oil droplets increases, resulting in larger, more floatable droplets.

The approach of the pH effect is illustrated in Fig. 3c. Fixing initial oil content concentration at 665 ppm, as pH increases from 6 to 7.5, the removal rate declines from 58.63% to 53.52%. Sharma et al.^62^ observed a similar trend for pH values between 5 and 7. Controversially, an additional increase of pH from 7.5 to 9 develops the rate from 53.52% to 60.29% as previously demonstrated in^63^.

Ammar et al.^64^ stated that the optimum pH value for the COD reduction rate is between 7.0 and 9.0. The rate of removal is influenced by the pH in terms of the solubility of Al(OH)_3_.

In the alkaline pH, the maximum removal efficiency is said to be consistently attained, which is related to the enhanced chemical dissolution rate of aluminum^65^.

At low pH (acidic pH), cationic monomeric species Al^3+^ and Al(OH)^2+^ are more prevalent^66^, and the solubility of Al(OH)_3(s)_ rises. The sparingly soluble hydrolyzed Al^3+^ products dissolved in the acidic solution cause a reduction in the amount of adsorbed and segregated pollutants. In addition to the massive size of H_2_ bubbles, which evolve from acidic solutions, they don’t float well^56^.

In contrast, at alkaline pH, the Al^3+^ and OH^-^ ions formed at the anode and cathode, respectively interact to generate a variety of monomeric species such as Al(OH)_2_^2+^, Al(OH)^2+^ as well as polymeric species as Al_6_(OH)_15_^3+^, Al_13_(OH)_34_^5+^, and Al_7_(OH)_17_^4+^. These gelatinous charged hydroxo-cationic complexes can successfully break the emulsion and adsorb pollutants to form neutralized charges. Through complicated polymerization/precipitation kinetics, these species eventually change into insoluble amorphous Al(OH)_3(s)_^66^. Additionally, the hydrogen bubbles are small, which enhances buoyancy^56^.

### Calcium removal rate experimental analysis

#### Fitting the model

**Fig. 4:**
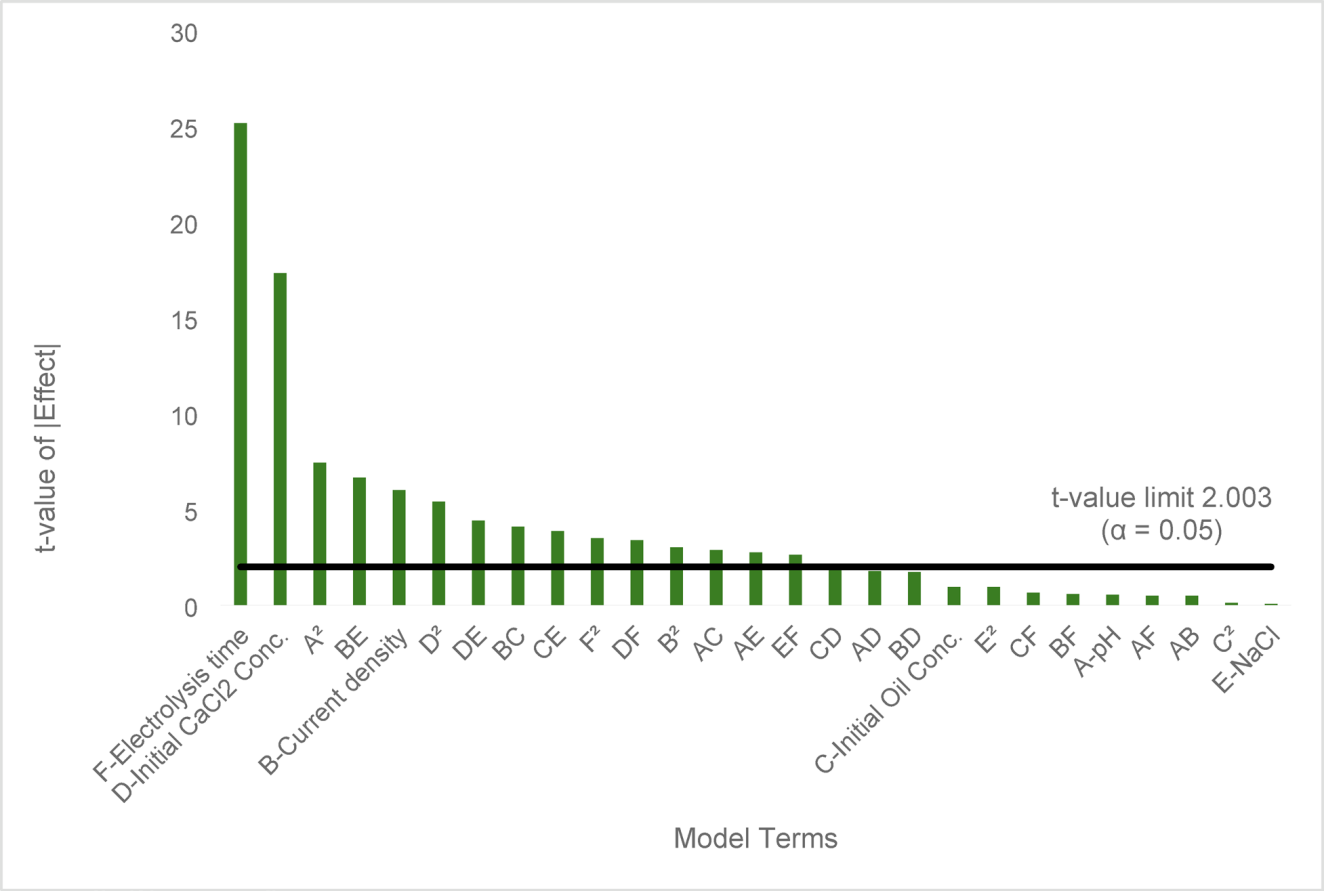
Pareto Chart for calcium removal rate.

Table 4 shows the findings of the quadratic regression model ANOVA performed for the calcium removal rate. The effective factors in this model are also represented as B, D, F, AC, AE, BC, BE, CE, DE, DF, EF, A2, B2, D2, and F2, as their *p*-values are less than 0.05. The Pareto chart (Fig. 4) for the independent variables is displayed, demonstrating that the electrolysis time is the most influential variable, followed by the initial calcium concentration.

**Table 4:** ANOVA for quadratic model for % Removal of calcium.

Source	Sum of Squares	df	Mean Square	*F*-value	*p*-value	
Model	23,213.49	27	859.76	47.78	< 0.0001	significant
A-pH	5.36	1	5.36	0.2977	0.5875	
B-Current density	649.61	1	649.61	36.10	< 0.0001	
C-Initial oil content conc	16.18	1	16.18	0.8990	0.3471	
D-Initial calcium conc	5399.18	1	5399.18	300.03	< 0.0001	
E-NaCl	0.0695	1	0.0695	0.0039	0.9507	
F- Electrolysis time	11,372.85	1	11,372.85	631.98	< 0.0001	
AB	4.33	1	4.33	0.2406	0.6257	
AC	149.64	1	149.64	8.32	0.0056	
AD	57.07	1	57.07	3.17	0.0804	
AE	137.37	1	137.37	7.63	0.0077	
AF	4.40	1	4.40	0.2445	0.6229	
BC	302.08	1	302.08	16.79	0.0001	
BD	54.16	1	54.16	3.01	0.0883	
BE	798.00	1	798.00	44.34	< 0.0001	
BF	6.11	1	6.11	0.3394	0.5625	
CD	61.79	1	61.79	3.43	0.0691	
CE	270.69	1	270.69	15.04	0.0003	
CF	7.74	1	7.74	0.4303	0.5145	
DE	350.50	1	350.50	19.48	< 0.0001	
DF	206.05	1	206.05	11.45	0.0013	
EF	124.38	1	124.38	6.91	0.0110	
A^2^	996.97	1	996.97	55.40	< 0.0001	
B^2^	163.44	1	163.44	9.08	0.0039	
C^2^	0.2708	1	0.2708	0.0150	0.9028	
D^2^	524.45	1	524.45	29.14	< 0.0001	
E^2^	16.09	1	16.09	0.8939	0.3485	
F^2^	220.72	1	220.72	12.27	0.0009	
Residual	1007.76	56	18.00			
Lack of Fit	939.76	47	19.99	2.65	0.0600	not significant
Pure Error	68.00	9	7.56			
Cor Total	24,221.24	83

The fit statistics in Table 5 show that the high value of R^2^ proves that independent parameters largely influence the response. How well the model predicts unknown data responses is indicated by the predicted R^2^ value. A closeness between R^2^, adjusted R^2^, and predicted R^2^ values is favored and is observed for the model. An insignificant lack of fit is also desired.

**Table 5:** Fit statistics of calcium removal rate model.

R^2^	0.9584
Adjusted R^2^	0.9383
Predicted R^2^	0.9024
Adeq Precision	31.4931

The proposed RSM model presents the relation between the calcium removal rate, and the actual operating variables as shown in Eq. (21)

21 $$\begin{gathered} \% Removal\;of\;calcium = + 181.23942 \, - \, 49.51069A \, - \, 1.07808B \, - \, 0.027272C \, - \, 0.101663D \hfill \\ + \, 14.92944E \, + \, 0.665356F \, + \, 0.101307AB \, + \, 0.004342AC \, - \, 0.004439AD \, - 0.666991AE \hfill \\ - 0.004712AF - \, 0.005289BC \, - \, 0.003707BD \, - 1.37807BE \, + \, 0.004759BF \, - \, 0.000029CD \hfill \\ + \, 0.005852CE \, + 0.000039 \, CF \, - \, 0.011022DE \, - \, 0.000334DF \, + \, 0.025004EF \, \hfill \\ + \, 3.43883A^{2} + 1.21291B^{2} + \, 2.46*10^{ - 6} C^{2} + \, 0.000300D^{2} - \, 0.609585E^{2} - \, 0.002757F^{2} \hfill \\ \end{gathered}$$

#### Influence of the operating parameters

##### Current density/initial oil content concentration

Increasing the current density efficiently affects the removal rate, which rises from 28.04% and 33.4% at 2.63 mA/cm^2^ to approximately 38.79% and 35.34% at 6.13 mA/cm^2^, while maintaining initial oil content concentration at 185 ppm and 665 ppm, respectively. However, for the higher oil content concentration of 665 ppm, a slight reduction in the removal rate of about 8% was observed before the subsequent increase. The main reason is that the low current density causes poor coagulation production, which inhibits the simultaneus adsorption of the high oil content and calcium concentrations. The removal rate is then improved by the subsequent increase in current, which generates sufficient adsorbents and effectively influence the electrocogulation.

**Fig. 5:**
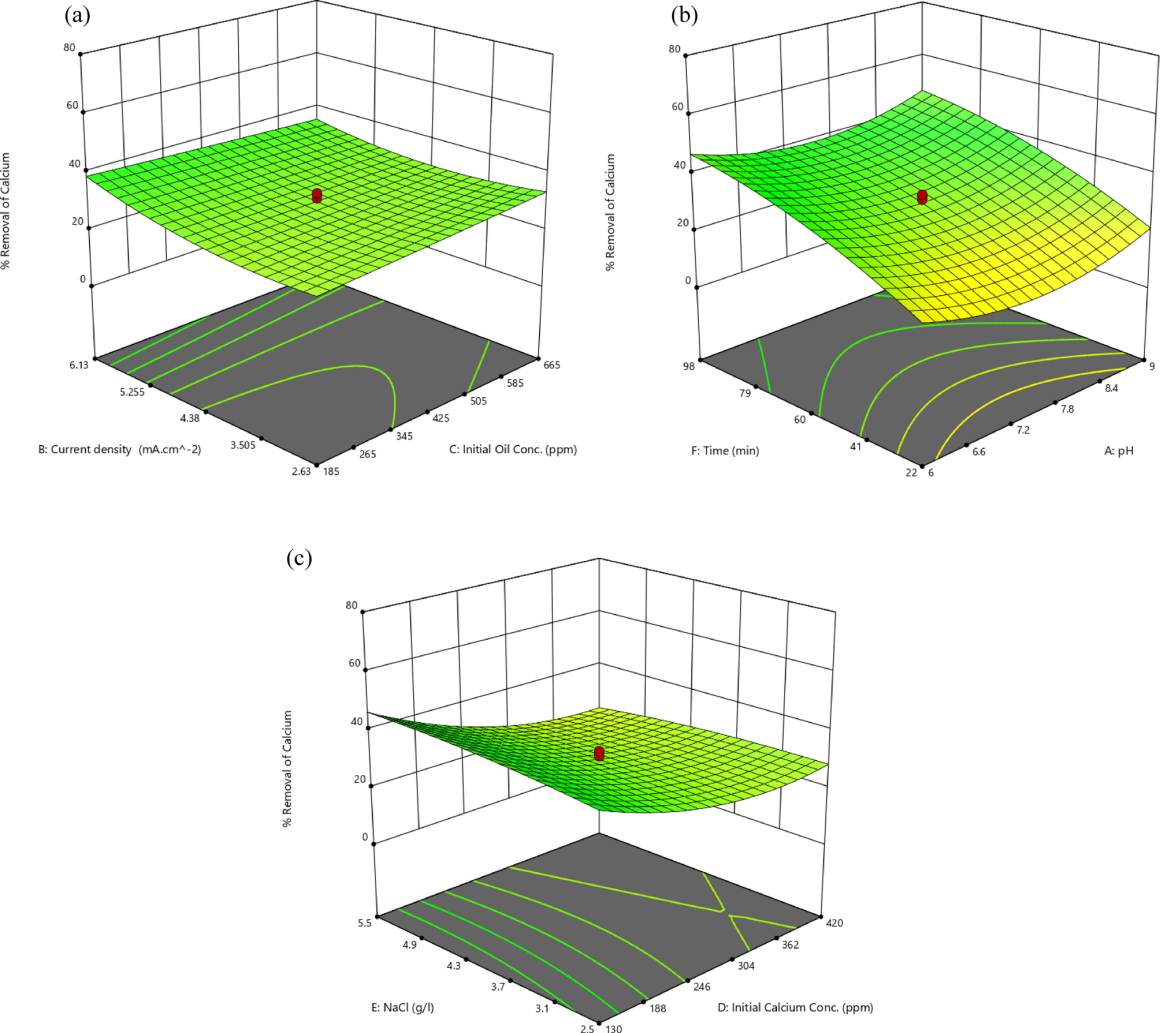
3D surface plots for calcium removal rate (**a**) current density and initial concentration of oil content (**b**) electrolysis time and pH (**c**) Initial concentrations of both calcium and NaCl.

The response behavior depicted in Fig. 5a is comparable to that described in^67^, where the oil content concentration was 523 ppm and was related to the uncontrolled liberation of various ions from the electrodes due to redox reactions taking place during the treatment, where these salts remained in suspension and neither adsorb nor float..

Figure 5a reveals that an increase in initial oil content from 185 to 665 ppm resulted in a gradual decrease in calcium removal rate. Maximum decline of about 9% was attained at the constant current density of 6.13 mA/cm^2^. Sefatjoo et al.^27^ proved the same trend.

##### Electrolysis time/pH

Electrolysis time is the most influential variable affecting the calcium removal rate (Fig. 4). Raising the reaction time from 22 to 98 min elevates the removal rate. Specifically, it rises from 20.16% to 46.91% at pH 6, and from 21.263% to 46.94% at pH 9.

As shown in Fig. 5b, the impact of pH on the calcium removal rate is similar to its effect on the oil content removal rate (section “Initial oil content concentration /pH”). When the pH is raised from 6 to 9, at a reaction time of 22 min, the removal rate initially drops by 35.7% before subsequently increasing by 64%.

##### Initial concentrations of both calcium and NaCl

The negative impact of raising the initial calcium concentration from 130 to 420 ppm on its removal rate is well documented. As shown in Fig. 5c, at a lower concentration of NaCl (2.5 g/l), the removal rate decreases by 32% while at a higher concentration of NaCl (5.5 g/l), the reduction is 50%. This phenomenon is primarily attributed to the fact that, under constant variables, the anodically dissolved Al^3+^ and H_2_ production rate remain constant and distributed across a large number of pollutants leading to the depletion of the adsorption capacity of hydroxide compounds^68^.

The negative consequence of raising the initial calcium concentration on both removal rates was explained by Sefatjoo et al.^27^ due to a deficiency of complexion sites relative to aqueous calcium.

The concentration of NaCl affects the removal rate depending on the initial concentration of calcium_._ This explains that their interaction is one of the most dominant variables in the model. For illustration, when calcium concentration is maintained at 130 ppm, increasing NaCl concentration from 2.5 g/l to 5.5 g/l boosts the removal rate from 41.7% to 46.42%. This is primarily due to enhanced conductivity, where sufficient chlorine act as an antipassive agent.

Conversely, when the concentration of calcium reaches 420 ppm, the same increase in NaCl concentration drops the rate from 28.4% to 23.51%. This decline is mainly attributed to the growing number of coagulants resulting from increased electrical conductivity^58^. Too much aluminum dissolution occurs at high NaCl and CaCl_2_ concentrations, leading to an undesired interaction between the coagulant and the pollutant^69^**.** By increasing both concentrations, the excess amount of Cl^−^ ion reacts with Al(OH)_3_. As a result, unstable substances like Al(OH)_2_Cl, Al(OH)Cl_2_, and AlCl_3_ are produced. Ultimately, these molecules transform into the AlCl_4_^−^form, which has a minimal adsorption impact. As a result, the quantity of coagulants and, consequently, the removal rate are reduced^70^.

### Optimization and validation of the EC process

It is obvious that interactions are so impactful in models. No variable gives one response on its own. Thus, optimization must be carried out to maximize the removal rates of both pollutants while the other independent variables are kept within the range.

The highest desirability was chosen to verify the experimental conditions. The optimum levels of the variables were selected using the regression analysis of the model equation for both removals and the results are presented in Table 6.

**Table 6:** The optimum number of variables and predicted removal rates.

pH	Current density (mA/cm^2^)	Initial oil content conc (ppm)	Initial calcium conc (ppm)	NaCl Conc (g/l)	Electrolysis time (min.)	% Predicted Oil Content Removal	% Predicted Calcium Removal	Desirability
9	6.123	588	130	2.5	98	91.274	72.892	0.955

Additional experiments were carried out to validate the removal rates under the calculated optimum conditions generated by the software. Compared to the predicted intervals (91.274 for oil content removal rate and 72.892 for calcium removal rate), the average response’s confirmation sample is % Oil Content Removal = 89.5377% and % of Calcium Removal = 75%. The model is confirmed and validated since the average responses of the confirmation experiment fall within the confirmation node’s prediction interval.

### Energy consumption and cost analysis

To ascertain the viability and the feasibility of the EC technique, it is necessary to calculate the energy consumption and the total operating cost. The values of the electrical energy consumption (E) and total cost (TOC) calculated at each location were fitted to a quadratic model using RSM, with high R^2^ values of 98.08% and 98.11%, respectively. The correlation between operating variables, electrical energy consumption, and overall cost is shown by Eq. (22–23).

22 $$\begin{gathered} E = 10.16544{-}1.94878A{-}0.57044B{-}0.001842C{-}0.004057D \hfill \\ {-}0.594887E \, {-} \, 0.022801F \, {-} \, 0.000732AB \, + \, 0.000148AC \, + \, 0.000515AD \hfill \\ \, + \, 0.022666AE \, {-} \, 0.000897AF \, + \, 0.000250BC \, {-} \, 0.000156BD \, {-} \, 0.138134BE \hfill \\ \, + \, 0.022989BF \, {-} \, 0.00000104035CD \, + \, 0.000466CE \, + \, 0.000000333661CF \, \hfill \\ + \, 0.000157DE \, {-} \, 0.000016DF \, {-} \, 0.003284EF \, + \, 0.112037A^{2} \, + \, 0.116132B^{2} \, \hfill \\ {-} \, 0.00000191012C^{2} \, + \, 0.00000148401D^{2} \, + \, 0.096838E^{2} \, + \, 0.000125F^{2} \hfill \\ \end{gathered}$$

23 $$\begin{gathered} TOC = 8.58859 \, {-} \, 1.65023A \, {-} \, 0.478017B \, {-} \, 0.001631C \, {-} \, 0.003360D \, \hfill \\ {-} \, 0.497741E \, {-} \, 0.019149F \, {-} \, 0.001133AB \, + \, 0.000129AC \, + \, 0.000431AD \, \hfill \\ + \, 0.018711AE \, {-} \, 0.000777AF \, + \, 0.000216BC \, {-} \, 0.000138BD \, {-} \, 0.117932BE \, \hfill \\ + \, 0.019723BF \, {-} \, 0.000000845777CD \, + \, 0.000400CE \, + \, 0.000000388409CF \, \hfill \\ + \, 0.000128DE \, {-} \, 0.000014DF \, {-} \, 0.002807EF \, + \, 0.095137A^{2} \, + \, 0.098616B^{2} \, \hfill \\ {-} \, 0.00000162807C^{2} \, + \, 0.00000124891D^{2} \, + \, 0.082123E^{2} \hfill \\ \end{gathered}$$

At the optimum conditions mentioned in Table 6, the calculation of E = 12 kWhm^-3^ and the TOC = 10.32 EGPm^-3^.

### Sludge characterization and its utilization

Following the optimized experimental procedures, the resultant sludge as well as the scum was dried and subsequently analyzed using FTIR, EDX and SEM characterization.

#### FTIR characterization

**Fig. 6:**
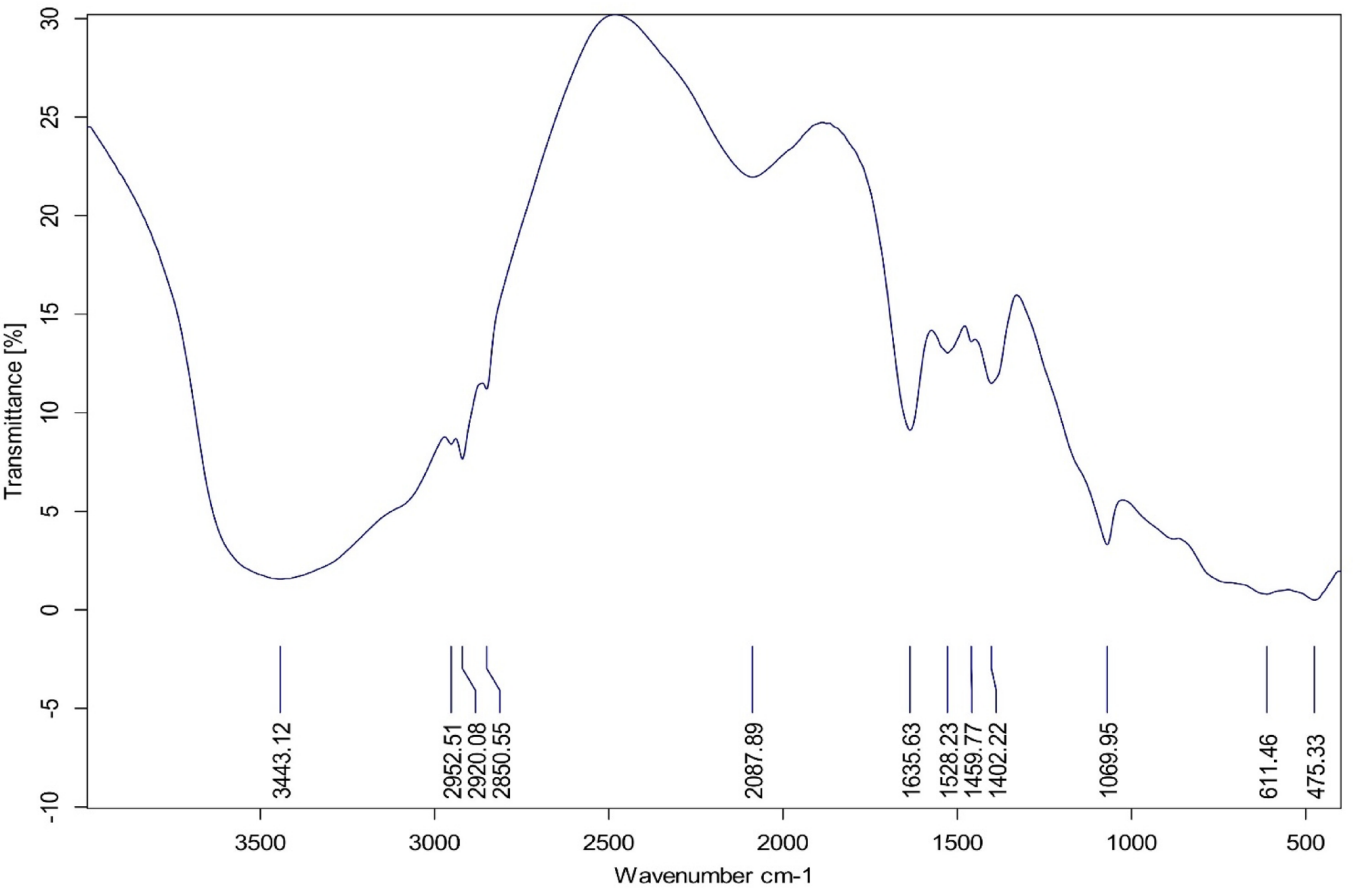
FTIR spectra of the produced sludge and scum at the optimum conditions.

Fourier Transform Infrared spectroscopy (FTIR) is used to determine the functional groups in the produced sludge and the scum. As shown in Fig. 6, variations in the infrared absorption bands (500–4000 cm^−1^) shows the interactions taking place between surface sites and solute species. The spectrum displays vibrations characteristic of the –OH group, seen as a prominent broad peak at 3443.12 cm^−1^^71^. C-H stretching vibrations, which indicate aliphatic compounds cause specific peaks at 2952.51, 2920.08, and 2850.55 cm^−1^^72,73^. Moreover, bands observed at 2087.89 cm^−1^ shows the existence of C≡C bonds^74^. Arising from H–O-H bending vibrations, the IR band at 1635.63 cm^−1^ signifies the existence of surface-adhered water on ≡Al_y_(OH)_3x_^75,76^. Furthermore, the band at 1528.23 cm^−1^ suggests the presence of surface ≡AlOH bending vibrations^26^. This band also is related to C = C stretching in aromatic compounds. Peaks found at 1459.77 and 1402.22 cm^−1^ correspond with aliphatic compounds, C-H bending vibrations^77^. Absorptions within the range of 500–1100 cm^−1^ signify the presence of aromatics with associated oxides^71^. Specifically, the band at 1069.95 cm^−1^ relates to the formation of gelatinous ≡ Al_y_(OH)_3×_ colloids^75^. These results show the crucial role of the generated sludge and scum functional groups in the removal of pollutants.

#### EDX characterization

**Fig. 7:**
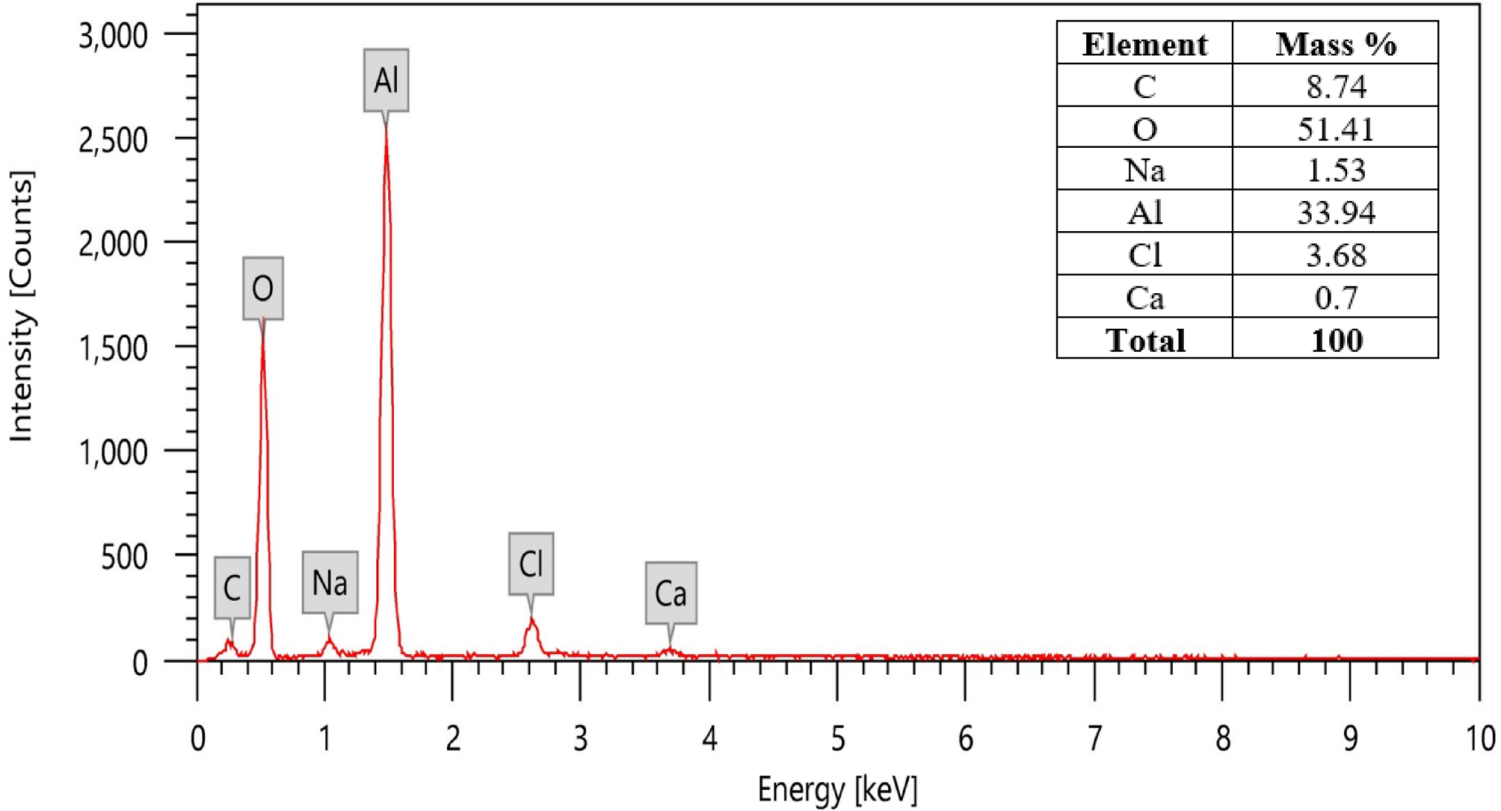
EDX analysis of the produced sludge and scum at the optimum conditions.

Energy-dispersive X-ray spectroscopy (EDX), shown in Fig. 7, was used to establish the elemental composition of the produced sludge and scum. Analysis reported the presence of C, Cl, Al, Na, O, and Ca. High levels of aluminum and oxygen were discovered particularly, thought to be caused by the oxides and hydroxides of aluminum formed as coagulants by the dissolving of aluminum electrodes. The observed chlorine is probably connected to the production of Cl_2_, HClO, and OCl^−^ as discussed previously (section “Initial concentrations of both calcium and NaCl”) as well because of the added NaCl and CaCl_2_. Gousmi et al.^36^ has reported comparable findings.

#### SEM characterization

**Fig. 8:**
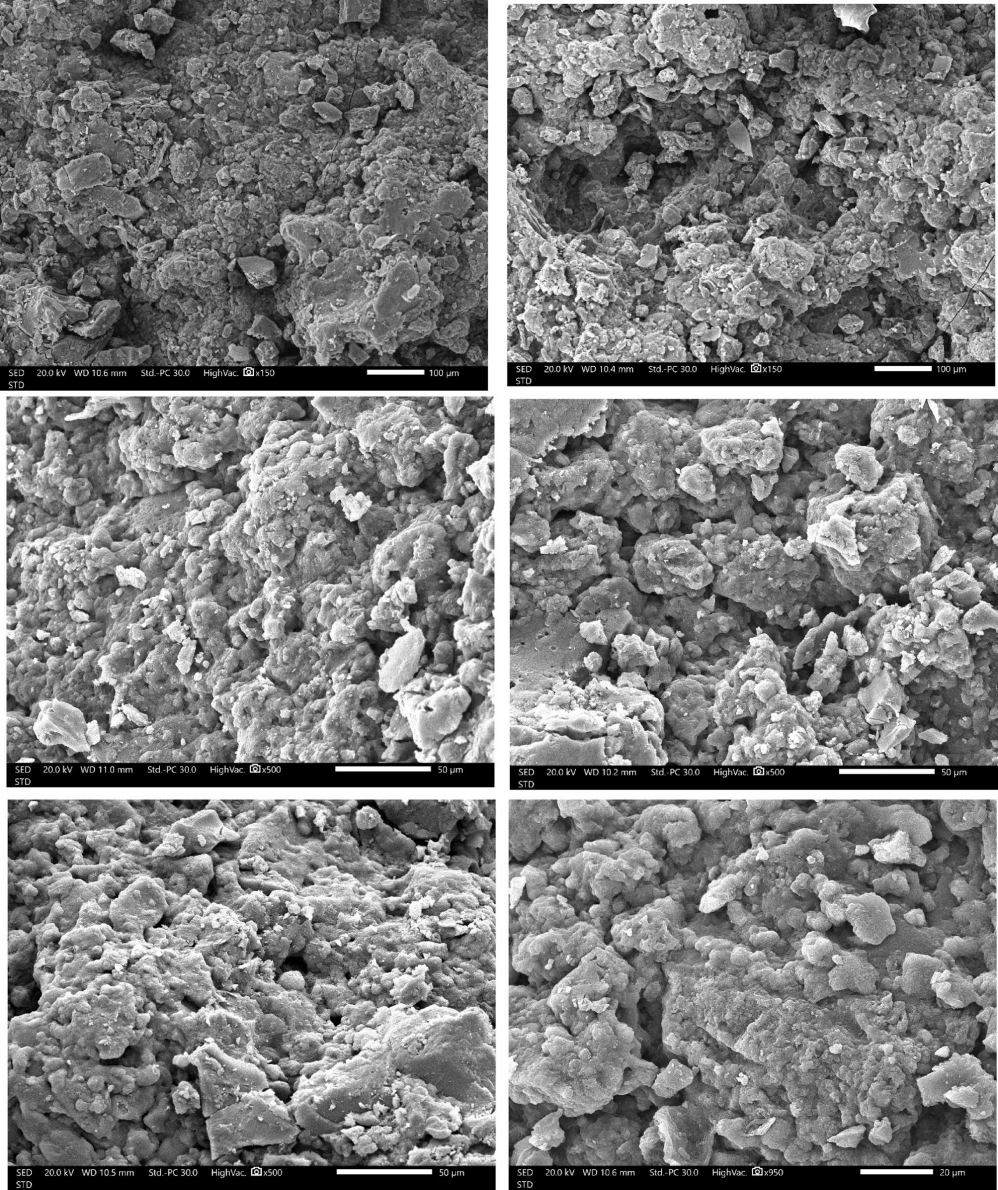
SEM images of the produced sludge and scum at the optimum conditions.

Scanning electron microscopy (SEM) images (Fig. 8) show heterogeneous sludge and scum morphology, characterized by high surface area, porosity, and nonuniform organic–inorganic complex composition. Fine particles adhere to larger conglomerates, which look like aluminum hydroxide agglomerates. Precipitation during electrocoagulation process probably yields these finer particles when destabilized oil droplets and dissolved calcium ions coprecipitate with the hydroxide forms of the coagulant metals.

The results of FTIR, EDX, and SEM analysis show that by incorporating the sludge into the soil, the presence of Ca, Na, and Al enhances the physicochemical qualities of the sludge, which may ultimately contribute to improving soil fertility^78^. The sludge’s Al content may also be collected and utilized to treat wastewater^79^. Additionally, Stendahl et al.^80^ discovered that aluminum can be extracted from the impurities in an alum sludge and reused as a coagulant in the water purification process.

### COMSOL simulation

All the equations necessary to solve the simulations are input into the COMSOL program after the geometry of the model has been added. The electrolyte’s conductivity was measured at the optimum condition to be 5.95 mS/cm, which falls within the reported conductivity ranges of typical PRW (2–7 mS/cm) mentioned in^36^. Using numerical solutions, COMSOL addresses these equations. The results will be retrieved after solving.

**Fig. 9:**
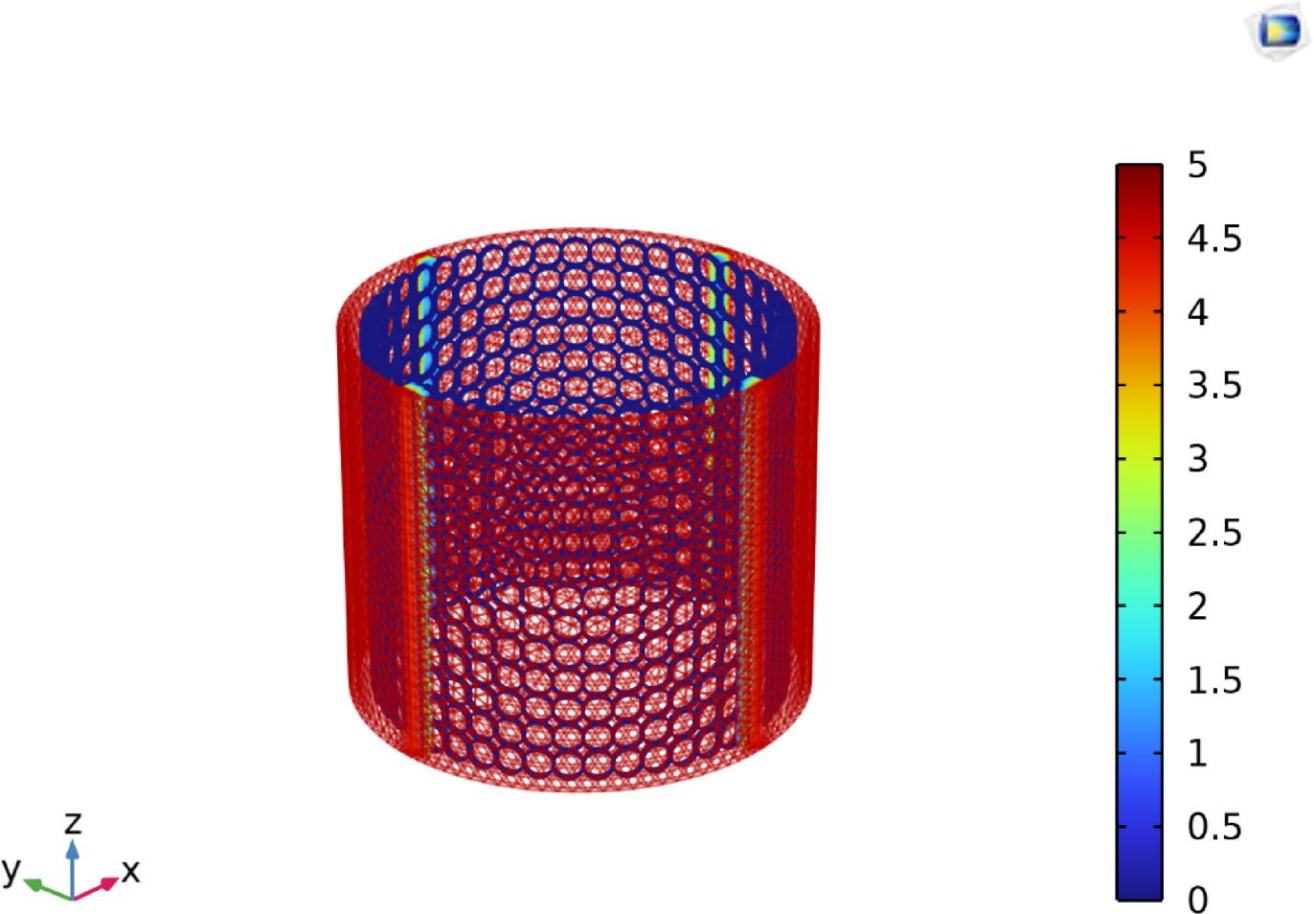
Voltage distribution at electrodes across the EC Cell.

Figure 9 shows the voltage distribution among the used cell. It shows that the voltage is highest on the anode surface and gradually drops until it reaches zero on the cathode surface.

**Fig. 10:**
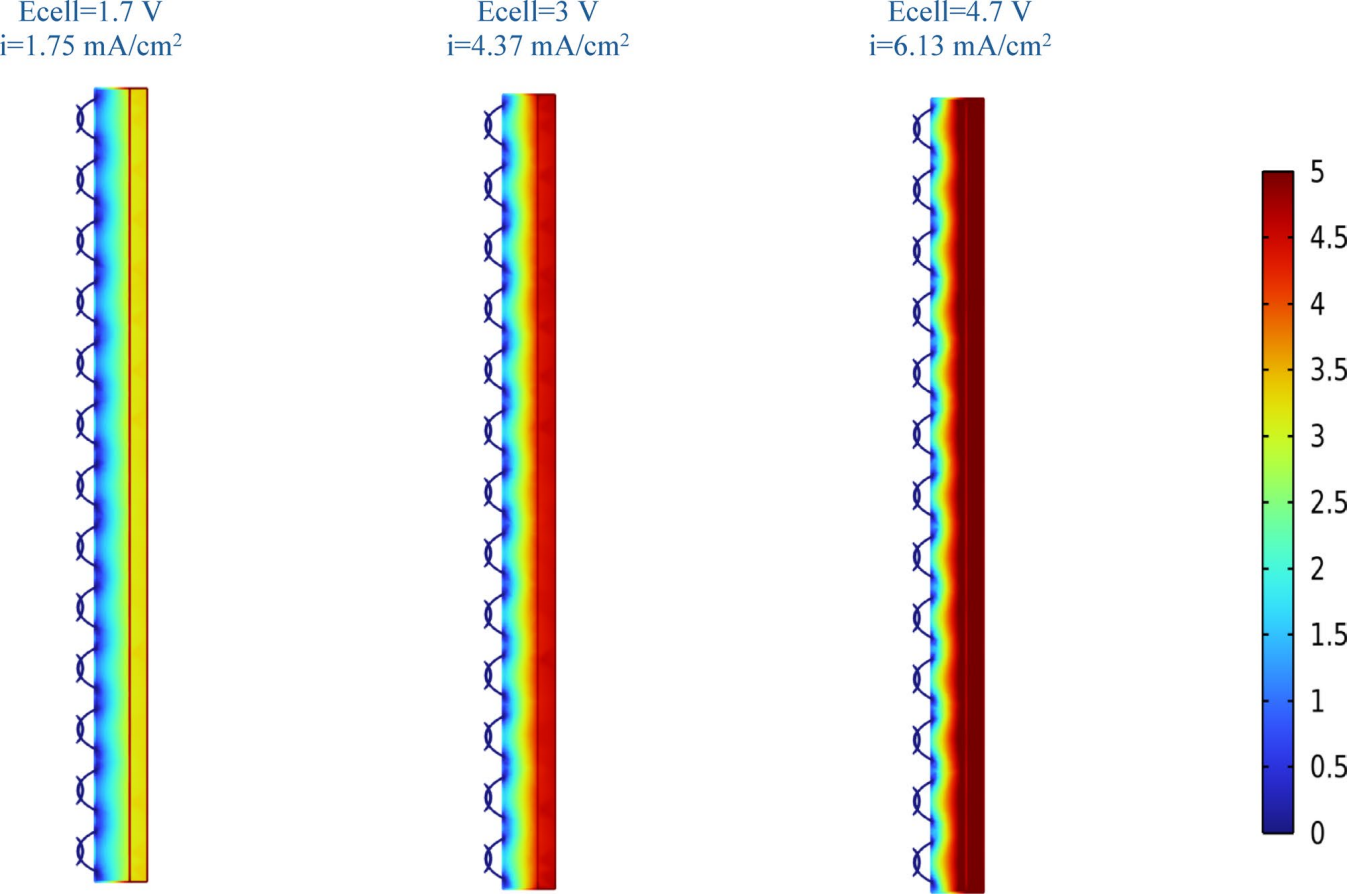
Distribution of the voltage across the EC reactor at various applied voltages.

The distribution in Fig. 10 is more illustrative and thorough, showing the highest voltage at the anode on the right and the lowest voltage at the cathode on the left. The distribution at three different voltages is also depicted in Fig. 10. The distribution is clearly more uniform at the maximum voltage (4.7 V), which corresponds to the highest current density (6. 13 mA/cm^2^), which explains why the higher the voltage and current density, the higher the removal rate, not just at the electrode surface but throughout the electrolyte, resulting in a higher reaction rate.

## Conclusion

This research studied the electrocoagulation technique for treating petroleum refinery wastewater, using Response Surface Methodology to analyze the relationships between independent variables and observed responses. A Central Composite Design was applied to identify the experimental scope, leading to the suggestion of a quadratic model for each response. ANOVA analysis showed strong correlations with high R^2^ values for the developed models: 0.9033 for oil content removal efficiency, 0.9584 for calcium removal efficiency, 0.9808 for energy consumption, and 0.9811 for total operating cost. Three-dimensional graphs visualized the impact of different factors on these responses, highlighting electrolysis time as the most significant factor positively influencing both removal rates. Conversely, initial calcium concentration negatively affected both rates. A pH increase from 6 to 7.5 initially reduced removal rates, but further elevation to 9 enhanced both efficiencies. Calcium removal efficiency was improved by decreasing the initial oil content concentration, increasing the current density, and adjusting NaCl concentration based on the calcium concentration levels. Furthermore, the removal of oil content was enhanced by raising initial oil content concentration, decreasing NaCl concentration, and increasing current density at high reaction electrolysis time. Therefore, the maximum oil content removal rate of 91.3% and a calcium removal rate of 72.9% were achieved at the estimated optimum conditions to be a pH of 9, a current density of 6.123 mA/cm^2^, an initial calcium concentration of 130 ppm, an initial oil content concentration of 588 ppm, a NaCl concentration of 2.5 g/l, and a total electrolysis time of 98 min. Concurrently, energy consumption was estimated to be 12 kWh/m^3^, with total operating costs of 10.32 EGP/m^3^, approximately US$0.21 per m^3^, which is notably lower than the estimates reported by Sefatjoo et al.^27^. At the optimum operating conditions, electrolysis time of 35.5 min, current density of 3.85 mA/cm^2^, initial calcium concentration of 250 mg/L, and initial turbidity of 85 NTU, the system reached calcium and turbidity removal rates of 36% and 93.5%, respectively, at a total cost of US $1.58/m^3^. COMSOL software validated the positive influence of current density in the electrocoagulation process, reinforcing electrocoagulation as a viable alternative for oil refinery wastewater treatment. The simple reactor configuration and the use of commercially available aluminum electrodes support the scale-up potential of the proposed technique. The favorable sludge characteristics and potential reuse options, together with the high removal efficiencies for oil content and calcium ions achieved at low operating costs are comparable to those reported for other EC systems treating real petroleum wastewaters, as documented by Shokri and Fard^81^ and Nigri et al.^82^, thereby emphasizing the practical applicability and the economic feasibility of the proposed technique.

## Supplementary Information

Below is the link to the electronic supplementary material.

Supplementary Material 1

## Data Availability

All data used during this study are included in this published article and its supplementary information files.
