# Supplementary material for: Process modeling and sludge characterization of electrocoagulation for the removal of oil-in-water emulsions and calcium from petroleum refinery wastewater
Source: Sci Rep. 2026 Mar 3;16:7954. doi: 10.1038/s41598-026-37854-8 (PMC12957336; doi:10.1038/s41598-026-37854-8)
Supplement: Supplementary file 1 — Supplementary Material 1 [file 41598_2026_37854_MOESM1_ESM.docx]

**Process Modeling and Sludge Characterization of Electrocoagulation for Oil-in-Water Emulsion and Calcium Removal from Petroleum Refinery Wastewater**

Yomna E. Mohamed^a^, Dina A. El-Gayar^a^, Nevine K. Amin^a^ and E-S. Z. El-Ashtoukhy^a, *^

^a^ Chemical Engineering Department, Faculty of Engineering, Alexandria University, Alexandria, Egypt.

*Corresponding author. E-mail: el.elashtoukhy@alexu.edu.eg

Supplementary Table S1: The experimental runs of CCD through the EC process.

|  | **Variables** | | | | | | **Results** | | | |
| --- | --- | --- | --- | --- | --- | --- | --- | --- | --- | --- |
| **Run** | **pH** | **Current density**  **(mA/cm^2)** | **Initial oil content conc. (ppm)** | **Initial calcium conc. (ppm)** | **NaCl concentration**  **(ppm)** | **Electrolysis time (min.)** | **Oil Content Removal Rate (%)** | | **Calcium Removal Rate (%)** | |
|  |  |  |  |  |  |  | **Experimental Values** | **Predicted Values** | **Experimental Values** | **Predicted Values** |
| 1 | 6 | 2.62476 | 665 | 420 | 5.5 | 22 | 56.16 | 51.91 | 25.00 | 24.23 |
| 2 | 9 | 6.12445 | 185 | 130 | 2.5 | 98 | 75.10 | 83.33 | 71.43 | 74.50 |
| 3 | 6 | 6.12445 | 665 | 420 | 2.5 | 22 | 54.27 | 52.60 | 25.00 | 23.54 |
| 4 | 9 | 2.62476 | 185 | 420 | 2.5 | 22 | 45.41 | 46.13 | 23.00 | 20.64 |
| 5 | 7.5 | 4.37461 | 425 | 275 | 2 | 60 | 81.43 | 80.76 | 30.50 | 27.67 |
| 6 | 6 | 2.62476 | 185 | 420 | 5.5 | 98 | 63.47 | 63.60 | 50.00 | 45.01 |
| 7 | 7.5 | 6.99937 | 425 | 275 | 4 | 60 | 77.95 | 73.14 | 42.86 | 43.14 |
| 8 | 6 | 2.62476 | 185 | 420 | 5.5 | 22 | 52.70 | 47.73 | 16.67 | 20.43 |
| 9 | 7.5 | 4.37461 | 425 | 500 | 4 | 60 | 27.28 | 30.48 | 33.33 | 31.20 |
| 10 | 6 | 2.62476 | 665 | 130 | 2.5 | 98 | 61.59 | 63.08 | 60.00 | 52.99 |
| 11 | 9 | 6.12445 | 665 | 130 | 2.5 | 98 | 84.63 | 92.28 | 75.00 | 72.69 |
| 12 | 7.5 | 4.37461 | 425 | 275 | 4 | 60 | 53.00 | 51.14 | 26.99 | 30.06 |
| 13 | 6 | 6.12445 | 665 | 130 | 2.5 | 98 | 86.99 | 87.19 | 60.00 | 64.07 |
| 14 | 6 | 2.62476 | 665 | 130 | 2.5 | 22 | 53.43 | 55.56 | 20.00 | 25.34 |
| 15 | 6 | 2.62476 | 185 | 130 | 5.5 | 98 | 63.77 | 65.63 | 66.67 | 65.78 |
| 16 | 7.5 | 4.37461 | 425 | 275 | 4 | 120 | 42.14 | 37.77 | 43.00 | 40.83 |
| 17 | 6 | 6.12445 | 665 | 420 | 5.5 | 22 | 45.00 | 51.78 | 20.00 | 15.81 |
| 18 | 6 | 2.62476 | 185 | 420 | 2.5 | 98 | 57.34 | 59.11 | 36.24 | 41.00 |
| 19 | 6 | 6.12445 | 185 | 130 | 2.5 | 98 | 77.75 | 79.54 | 75.00 | 72.13 |
| 20 | 6 | 6.12445 | 665 | 130 | 5.5 | 22 | 56.00 | 52.51 | 33.33 | 37.00 |
| 21 | 9 | 2.62476 | 665 | 130 | 5.5 | 22 | 58.29 | 55.10 | 38.00 | 44.29 |
| 22 | 9 | 6.12445 | 185 | 130 | 5.5 | 22 | 41.20 | 45.07 | 40.00 | 35.51 |
| 23 | 6 | 2.62476 | 665 | 130 | 5.5 | 98 | 55.00 | 62.09 | 80.00 | 75.02 |
| 24 | 9 | 6.12445 | 185 | 130 | 2.5 | 22 | 58.92 | 53.04 | 42.86 | 48.08 |
| 25 | 7.5 | 4.37461 | 425 | 275 | 4 | 60 | 50.01 | 51.14 | 26.00 | 30.06 |
| 26 | 9 | 2.62476 | 185 | 130 | 2.5 | 98 | 70.75 | 69.22 | 50.00 | 53.48 |
| 27 | 7.5 | 4.37461 | 425 | 275 | 4 | 60 | 52.50 | 51.14 | 30.00 | 30.06 |
| 28 | 9 | 6.12445 | 665 | 130 | 5.5 | 98 | 84.60 | 87.14 | 80.00 | 74.24 |
| 29 | 7.5 | 4.37461 | 425 | 275 | 4 | 60 | 45.00 | 51.14 | 29.60 | 30.06 |
| 30 | 6 | 6.12445 | 185 | 420 | 5.5 | 98 | 75.17 | 74.13 | 51.00 | 46.74 |
| 31 | 9 | 2.62476 | 665 | 130 | 2.5 | 98 | 72.01 | 67.48 | 60.00 | 60.55 |
| 32 | 6 | 2.62476 | 185 | 130 | 2.5 | 98 | 66.46 | 66.13 | 49.00 | 52.18 |
| 33 | 9 | 6.12445 | 185 | 420 | 2.5 | 22 | 35.68 | 37.51 | 35.49 | 36.63 |
| 34 | 7.5 | 4.37461 | 425 | 50 | 4 | 60 | 44.33 | 40.84 | 60.00 | 59.31 |
| 35 | 9 | 2.62476 | 185 | 130 | 5.5 | 98 | 62.70 | 66.08 | 60.00 | 61.08 |
| 36 | 6 | 2.62476 | 185 | 130 | 5.5 | 22 | 48.90 | 51.29 | 37.60 | 33.85 |
| 37 | 6 | 2.62476 | 665 | 420 | 2.5 | 98 | 61.84 | 60.26 | 33.33 | 37.80 |
| 38 | 5 | 4.37461 | 425 | 275 | 4 | 60 | 62.85 | 66.79 | 50.00 | 51.08 |
| 39 | 6 | 2.62476 | 185 | 130 | 2.5 | 22 | 61.68 | 55.10 | 20.00 | 25.95 |
| 40 | 9 | 6.12445 | 665 | 420 | 5.5 | 22 | 55.70 | 50.71 | 20.00 | 15.64 |
| 41 | 7.5 | 4.37461 | 425 | 275 | 4 | 60 | 56.00 | 51.14 | 27.27 | 30.06 |
| 42 | 7.5 | 4.37461 | 425 | 275 | 4 | 60 | 48.93 | 51.14 | 31.20 | 30.06 |
| 43 | 9 | 2.62476 | 665 | 420 | 2.5 | 22 | 43.64 | 52.10 | 20.00 | 22.26 |
| 44 | 9 | 2.62476 | 665 | 420 | 5.5 | 22 | 50.69 | 50.14 | 27.27 | 23.00 |
| 45 | 7.5 | 4.37461 | 50 | 275 | 4 | 60 | 41.18 | 43.83 | 30.00 | 29.63 |
| 46 | 7.5 | 4.37461 | 425 | 275 | 6 | 60 | 77.84 | 78.12 | 28.57 | 27.58 |
| 47 | 7.5 | 4.37461 | 425 | 275 | 4 | 0 | 0.0000 | 4.10 | 0.0000 | -0.5583 |
| 48 | 6 | 6.12445 | 665 | 420 | 2.5 | 98 | 85.32 | 83.00 | 47.90 | 45.11 |
| 49 | 6 | 6.12445 | 185 | 420 | 2.5 | 98 | 70.68 | 71.15 | 58.33 | 57.19 |
| 50 | 9 | 6.12445 | 665 | 420 | 2.5 | 22 | 58.80 | 54.17 | 25.00 | 29.37 |
| 51 | 6 | 2.62476 | 665 | 420 | 5.5 | 98 | 71.00 | 64.26 | 50.00 | 50.24 |
| 52 | 6 | 2.62476 | 185 | 420 | 2.5 | 22 | 44.91 | 46.55 | 27.13 | 22.12 |
| 53 | 9 | 6.12445 | 665 | 130 | 2.5 | 22 | 60.58 | 65.49 | 40.50 | 44.84 |
| 54 | 6 | 6.12445 | 665 | 130 | 2.5 | 22 | 57.44 | 58.32 | 40.00 | 35.15 |
| 55 | 10 | 4.37461 | 425 | 275 | 4 | 60 | 72.66 | 68.47 | 55.56 | 52.03 |
| 56 | 6 | 6.12445 | 185 | 130 | 5.5 | 22 | 38.00 | 41.84 | 40.90 | 38.07 |
| 57 | 9 | 2.62476 | 185 | 420 | 5.5 | 98 | 62.00 | 58.45 | 36.36 | 36.45 |
| 58 | 6 | 6.12445 | 185 | 420 | 2.5 | 22 | 35.80 | 37.24 | 34.75 | 37.05 |
| 59 | 7.5 | 4.37461 | 425 | 275 | 4 | 60 | 45.99 | 51.14 | 33.00 | 30.06 |
| 60 | 9 | 6.12445 | 185 | 130 | 5.5 | 98 | 86.43 | 78.68 | 70.00 | 67.63 |
| 61 | 9 | 6.12445 | 185 | 420 | 5.5 | 98 | 66.00 | 69.67 | 35.60 | 39.24 |
| 62 | 9 | 2.62476 | 185 | 420 | 2.5 | 98 | 57.30 | 56.60 | 41.67 | 38.44 |
| 63 | 7.5 | 4.37461 | 425 | 275 | 4 | 60 | 54.00 | 51.14 | 25.00 | 30.06 |
| 64 | 9 | 2.62476 | 185 | 420 | 5.5 | 22 | 47.00 | 44.67 | 9.09 | 12.95 |
| 65 | 9 | 2.62476 | 185 | 130 | 2.5 | 22 | 62.20 | 60.28 | 33.33 | 28.33 |
| 66 | 9 | 2.62476 | 665 | 130 | 2.5 | 22 | 64.02 | 62.04 | 40.00 | 33.97 |
| 67 | 9 | 6.12445 | 665 | 420 | 2.5 | 98 | 88.77 | 82.49 | 50.00 | 49.87 |
| 68 | 9 | 2.62476 | 665 | 420 | 2.5 | 98 | 58.35 | 59.06 | 40.00 | 41.49 |
| 69 | 6 | 6.12445 | 185 | 130 | 2.5 | 22 | 48.60 | 47.16 | 50.00 | 44.64 |
| 70 | 6 | 6.12445 | 665 | 130 | 5.5 | 98 | 91.27 | 84.70 | 66.67 | 71.63 |
| 71 | 9 | 2.62476 | 185 | 130 | 5.5 | 22 | 49.30 | 53.83 | 28.91 | 30.22 |
| 72 | 9 | 6.12445 | 665 | 420 | 5.5 | 98 | 78.62 | 82.33 | 40.00 | 41.83 |
| 73 | 7.5 | 4.37461 | 425 | 275 | 4 | 60 | 49.90 | 51.14 | 28.10 | 30.06 |
| 74 | 6 | 6.12445 | 665 | 420 | 5.5 | 98 | 77.70 | 85.49 | 40.00 | 43.08 |
| 75 | 7.5 | 4.37461 | 425 | 275 | 4 | 60 | 55.00 | 51.14 | 32.60 | 30.06 |
| 76 | 6 | 6.12445 | 185 | 420 | 5.5 | 22 | 39.40 | 36.91 | 15.38 | 20.90 |
| 77 | 9 | 2.62476 | 665 | 420 | 5.5 | 98 | 54.33 | 60.41 | 45.45 | 47.93 |
| 78 | 7.5 | 4.37461 | 800 | 275 | 4 | 60 | 57.00 | 54.08 | 33.60 | 31.18 |
| 79 | 9 | 6.12445 | 665 | 130 | 5.5 | 22 | 55.70 | 57.04 | 40.00 | 40.70 |
| 80 | 9 | 2.62476 | 665 | 130 | 5.5 | 98 | 67.37 | 63.84 | 73.60 | 76.57 |
| 81 | 7.5 | 1.74984 | 425 | 275 | 4 | 60 | 58.16 | 62.66 | 37.00 | 33.69 |
| 82 | 9 | 6.12445 | 185 | 420 | 2.5 | 98 | 70.53 | 69.34 | 58.33 | 55.70 |
| 83 | 6 | 6.12445 | 185 | 130 | 5.5 | 98 | 81.42 | 77.54 | 66.67 | 71.27 |
| 84 | 6 | 2.62476 | 665 | 420 | 2.5 | 22 | 51.99 | 51.22 | 16.67 | 17.49 |
